# Phylogenetic Methods Meet Deep Learning

**DOI:** 10.1093/gbe/evaf177

**Published:** 2025-09-19

**Authors:** Svitlana Braichenko, Rui Borges, Carolin Kosiol

**Affiliations:** Institute of Genetics and Cancer, University of Edinburgh, Edinburgh EH4 2XU, UK; School of Mathematics and Statistics, Mathematical Institute, University of St Andrews, St Andrews KY16 9SS, UK; Institut für Populationsgenetik, Vetmeduni Vienna, Wien 1210, Austria; Centre for Biological Diversity, School of Biology, University of St Andrews, Fife KY16 9TH, UK

**Keywords:** machine learning, neural network, phylogenetics, phylodynamics and diversification studies

## Abstract

Deep learning (DL) has been widely used in various scientific fields, but its integration into phylogenetics has been slower, primarily due to the complex nature of phylogenetic data. The studies that apply DL to sequencing data often limit analyses to four-taxon trees. Many of these studies serve as “proof of principle” and perform similarly to traditional phylogeny reconstruction methods. New ways of using training data, such as encoding with compact bijective ladderized vectors or transformers, enable the handling of much larger trees and genomic data sets. This short perspective focuses on the application of DL in phylogenetics, introducing prevalent DL architectures. We highlight potential problems in the field by discussing the risks of using simulation-based training data and emphasize the importance of reproducibility and robustness in computational estimates. Finally, we explore promising research areas, including the combination of phylogenetics and population genetics in DL, the analysis of neighbor dependencies, and the potential to significantly reduce computational cost compared to traditional methods. This perspective illustrates the potential of DL in complementing traditional phylogeny reconstruction methods and aiding the advancement of phylogenetic analysis, especially in performing computationally demanding tasks such as model selection or estimating branch support values.

Significance We present a concise perspective on the intersection of phylogenetics—the study of evolutionary relationships between species or subspecies as manifested in phylogenetic trees—and artificial intelligence (AI). We primarily focus on how deep learning (DL), a subset of AI methods that particularly benefits from big data, can enhance the reconstruction and analysis of phylogenies in ecology and epidemiology. In light of the modern challenges of rapidly growing data, especially during pandemics, traditional methods such as maximum likelihood and Bayesian inference could be complemented by novel DL approaches. These approaches could also help us tackle challenges related to mismatches between simulated and empirical data, improve the robustness of inferences, and present promising directions for the future of the field, opening new directions for exploring phylogenetic data.

## Introduction

The advent of DL, a branch of machine learning (ML), has significantly transformed the analysis of genomic data sets (Schrider and Kern 2018). However, its adoption in phylogenetics has lagged due to challenges such as the unique structure of phylogenetic trees and the complexity of representing them in a manner suitable for DL algorithms (Penn et al. 2023). DL uses multilayered neural networks (NNs) to analyze complex data patterns. This short perspective discusses the application of DL in phylogenetics, highlighting the NN architectures used and recent advancements that have enabled their effective use in this field. We also extend our discussion to broader ML applications in phylogenetics, summarizing essential AI terminology in Table 1. For a full review of ML approaches in population genetics, refer to Schrider and Kern (2018). This concise perspective explores key studies in phylogenetic DL, as well as recent studies published after the last review by Mo et al. (2024) which we refer readers to for a comprehensive investigation.

**Table 1.** The glossary of ML terms. For introduction to ML, see Mohri et al. (2018)

Short term	Long term	Description
AI	Artificial intelligence	Intelligence of machines, particularly based on computer algorithms.
ML	Machine learning	Computational methods that use experience (training) to enhance performance or make accurate predictions (testing).
	Supervised learning	ML paradigm where input to the model is labeled corresponding to the desired outcome.
	Unsupervised learning	Unlike supervised learning, the model uses only unlabeled data.
	Classification	Algorithm assigns classes to the data.
	Regression	Algorithm predicts numerical values given some input data.
DL	Deep learning	Subfield of ML that uses NNs with multiple layers.
FFNNs	Feedforward neural networks	NNs have information flow in one direction, without feedback loops.
RNNs	Recurrent neural networks	NNs that include feedback connections.
CNNs	Convolutional neural networks	NNs process data with a known grid-like structure using convolution.
GNNs	Graph neural networks	NNs process data that can be represented as a graph.
GAN	Generative adversarial networks	ML framework containing a generator and discriminator to learn how to generate data that resembles a given training dataset.
NLP	Natural language processing	Use of languages by computers, biological sequence data can also be interpreted as a language.
LLM	Large language models	ML models acquiring knowledge about languages.
	Transformers	Standard architecture for building LLMs based on attention, meaning connection between different learning blocks.
SS	Summary statistics	Used to summarize set of observations, e.g. phylogenetic trees or sequencing data, in order to process large datasets.
CBL(D)V	Compact bijective ladderized (diversity-reordered) vector	Alternative to SS; tree encoding for preventing information loss.
	Bayesian optimization	Automated selection of the next hyperparameter from previously tested values, using a surrogate function.
DA	Domain adaptation	Training a model on one set of data and then using it on a related yet distinct set of data.
CQR	Conformalized quantile regression	Method generating support intervals that contain the true parameter value at a specified frequency (typically 95%).

For DL, please consult Goodfellow et al. (2016). NLP and related questions are presented by Jurafsky and Martin (2025). Hyperparameter optimization is well covered by Bischl et al. (2023). For more details about CQR, see Romano et al. (2019)

**Fig. 1.**
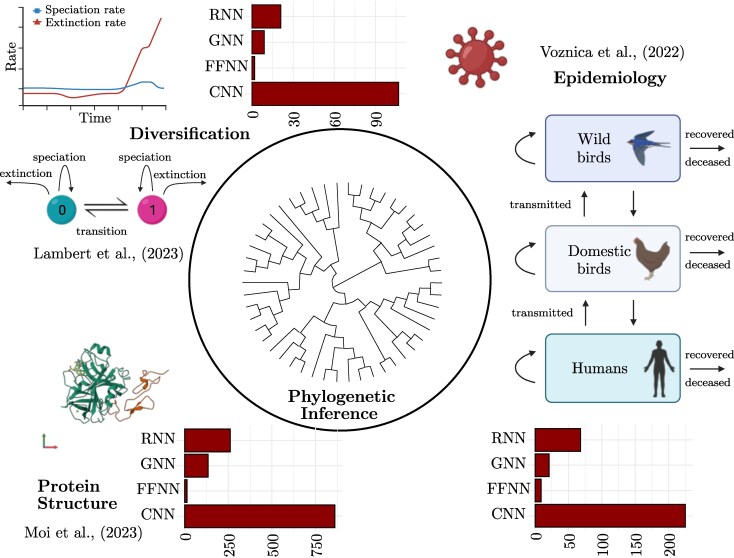
The number of studies using RNN, GNN, FFNN, and CNN in phylogeny, based on a PubMed full text search, is categorized according to their field of application: diversification, epidemiology, and protein structure. The search is performed with the keywords “Phylo* (field) Deep Learning (NN type)”. Each bar plot represents the frequency of the most commonly used NN architecture in each field. Examples of mathematical models used and studies using DL in some of the corresponding fields are also shown. Created in BioRender.com.

Compared to phylogenetics (Mo et al. 2024), population genetics has exercised DL earlier and more extensively (Schrider and Kern 2018). Both fields now primarily focus on supervised learning since the majority of training data, simulated from mathematical models, can be easily labeled to simplify learning tasks. The main applications of NNs in phylogeny, identified through this perspective and PubMed search, are summarized in Fig. 1.

## DL Methods in Phylogenetics

### DL Methods Improving Phylogeny Reconstruction

The progress in maximum likelihood and Bayesian inference for phylogeny reconstruction, driven by advanced statistical models and computational methods (Scornavacca et al. 2020; Stadler et al. 2024), has been significant. These methods enable high-accuracy inferences of evolutionary relationships but are limited by high computational costs (Lees et al. 2018) and sensitivity to model choice and dataset size (Lozano-Fernandez 2022). With genomic data production increasing rapidly, traditional methods struggle to handle large datasets are at risk of becoming rapidly obsolete. This has spurred interest in ML, particularly DL techniques, as an alternative approach to likelihood-based methods. DL not only manages large data volumes but may also enhance performance with dataset size. Although DL depends on the architecture and is often trained on simulated data, it can execute tasks without retraining, potentially cutting the computational costs of phylogenetic analysis. The use of a trained predictor, such as that by Suvorov et al. (2020), could significantly accelerate these processes.

**Fig. 2.**
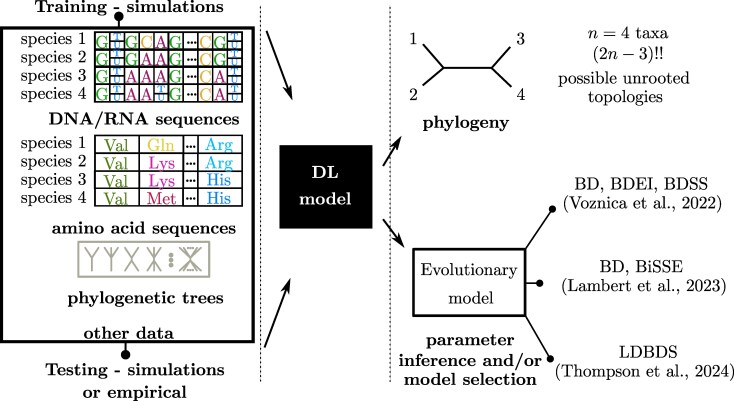
Workflow for DL models in phylogeny reconstruction: On the left-hand side, we illustrate the types of data used to train the DL model. Typically, this involves simulated data modeled within a specific range of evolutionary parameters. The same type of data is also employed for testing the model. While empirical data are most commonly used for this purpose, simulated data for selected parameters can also be utilized. It primarily consists of multiple sequence alignment (MSA) data, which includes sequences of DNA/RNA nucleotides or amino acids from multiple species. Some studies use images of species or ribosomal RNA, etc. The output from the DL model in most cases would be a predicted phylogeny. This is known as a notoriously difficult task due to the increase in the number of possible unrooted tree topologies ((2n−3)!!, where !! denotes the semifactorial) as the number of taxa, *n*, grows, resulting in an explosion of the parameter space. Some studies use a regression problem and along with predicted phylogeny allow for the inference of parameters in the underlying model or model selection. These studies often pretrain their DL models on simulated data from epidemiological models: birth–death (BD), birth–death with exposed-infectious individuals (BDEI), birth–death with superspreading (BDSS), location-dependent birth–death (LDBDS). And diversification models: BD, binary-state speciation and extinction (BiSSE).

Applying DL to infer phylogenies poses significant challenges, owing to the difficulty in encoding the hierarchical structure of phylogenetic trees (Penn et al. 2023). This challenge arises from the explosion in the number of possible unrooted tree topologies with the number of tips (detailed in Fig. 2). The vast model space also presents a challenge when constructing phylogenies. In this section, we briefly summarize the methods for inferring phylogenies, and we refer readers to Mo et al. (2024) for a comprehensive summary. Most of the DL applications in phylogenetics have focused on learning four-taxon trees, reducing the problem to a basic classification task with only three possible topologies. These DL models, typically trained on amino acid and protein sequences (Zou et al. 2020) and MSAs (Suvorov et al. 2020), including branch-length estimation (Suvorov and Schrider 2024), have shown promising results, particularly with noisy and incomplete alignments, outperforming maximum likelihood, maximum parsimony, and Bayesian inference (Suvorov et al. 2020). However, their performance on large-scale phylogeny reconstruction remains challenging, as Zaharias et al. (2022) found that NNs for quartet-based phylogeny estimation and combined via quartet amalgamation do not reach the accuracy of traditional methods on larger trees.

**Fig. 3.**
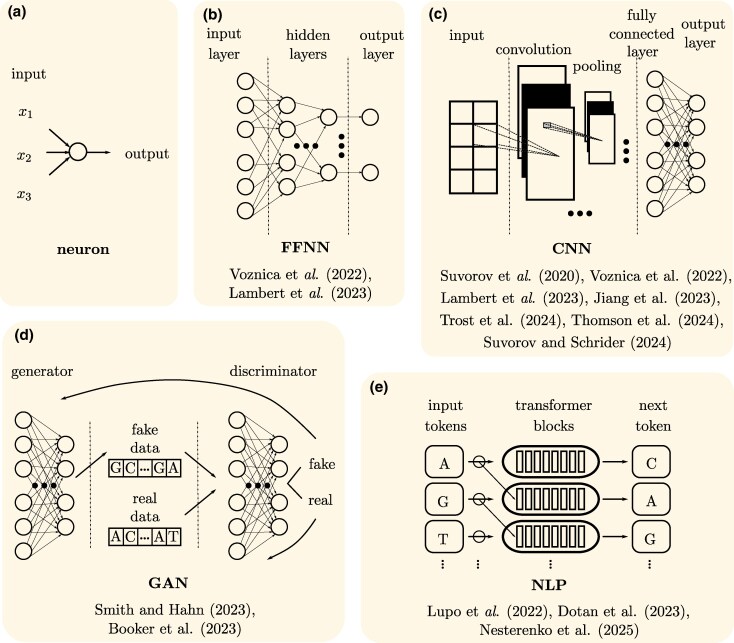
A model of neuron (a) and main types of DL models, explained in Table 1. The references reflect examples of studies that use corresponding model. In particular, Voznica et al. (2022) used FFNNs (b) with summary statistics (FFNN-SSs, explained in Table 1) and CNNs (c) with vectorial tree encoding called compact bijective ladderized vector (CNN-CBLV). This encoding allows the networks to directly interpret the trees’ structure, bypassing the limitation of generalizing tree reconstructions for variable size of taxa without the necessity to retrain the NN. Lambert et al. (2023) demonstrated how NNs can estimate diversification and extinction rates using techniques similar to those of Voznica et al. (2022), additionally incorporating complete diversity-reordered vector (CDV) tree representation. This model excels in handling complex scenarios like gene tree heterogeneity and incomplete lineage sorting, which are challenging for conventional methods. GANs (d) (Smith and Hahn 2023) excel in handling complex scenarios like gene tree heterogeneity and incomplete lineage sorting, which are challenging for conventional methods. The aspiration is that LLMs (e) would be able to cope with genome-wide or at least chromosome-wide alignments and patterns (e.g. Albors et al. 2025).

On the other hand, recent success in applying DL to phylodynamics datasets, such as viral genome sequences, have shown potential for enhancing epidemiological understanding. Unlike previous methods, NNs where trained on phylogenetic trees not only to estimate epidemiological parameters through regression but also for model selection via classification. Voznica et al. (2022) explored several NN architectures (see Fig. 3) and developed specialized encoding methods to transform large phylogenetic trees, typical for epidemiological models, into formats suitable for CNNs. Building on this, Lambert et al. (2023) developed DL models to analyze diversification dynamics from phylogenetic trees. Both studies employed CBLV (additionally CDV in Lambert et al. 2023) and various SSs. FFNN-SS and CNN-CBLV matched the accuracy of standard methods and offered significant speed-ups in epidemiology (Voznica et al. 2022). While CNN-CDV outperformed other architectures in diversification scenarios (Lambert et al. 2023) due to the absence of appropriate SSs for certain models (e.g. BiSSE), similar to those available in epidemiology (Saulnier et al. 2017). A major challenge remains the need to independently assess the best NN architecture and encoding technique for each new application. In conclusion, these findings mark initial steps in demonstrating DL’s potential as both an alternative and a complement to traditional phylogenetic and phylodynamic techniques, especially in scenarios requiring rapid analysis, such as during ongoing epidemic responses.

### Beyond Traditional Data Types and ML Frameworks

Interestingly, DL has been applied not only to sequence data but also to images of organisms and small genomic fragments. Elhamod et al. (2023) used used image data in CNNs for phylogenetic analysis by extracting morphological features from high-resolution species images to refine tree reconstructions. Moreover, Jiang et al. (2023) expanded the application of DL to incorporate ribosomal RNA and metagenomic data. Their method, DEPP, specifically addressed large genomic datasets to enhance the accuracy of placing new sequences onto existing phylogenetic trees.

Exploring a broader range of ML frameworks can offer new insights into phylogenetic reconstruction. PhyloGAN, developed by Smith and Hahn (2023) use GANs (see Fig. 3d) to infer phylogenetic relationships efficiently, exploring large and complex tree topologies, with less computational demand. However, it depends heavily on network architecture and requires accurate reflection of evolutionary diversity, affecting result accuracy. Recently, Nesterenko et al. (2025) demonstrated that Phyloformer, based on self-attention, similarly to transformers (Fig. 3e), matches traditional methods in accuracy and exceeds them in speed, and outperforms them under complex models. However, despite its proficiency in estimating evolutionary distances for large trees, it slightly trails in topological accuracy as sequence numbers increase. Similarly, the reinforcement learning approach to phylogenetic tree reconstruction by Azouri et al. (2024) provides advantages over traditional methods, such as avoiding local optima and efficiently managing large datasets. This method optimizes for long-term outcomes, potentially yielding more accurate global solutions. However, like phyloGAN, it depends heavily on the quality of training data. Additionally, setting up and training these models is complex, time-consuming, and lacks the interpretability of standard phylogenetic methods.

Lastly, we highlight the rising popularity of LLM approaches in phylogeny, thanks to their capacity to manage large genomic datasets. Lupo et al. (2022) and Dotan et al. (2023) use transformers (Fig. 3f) for aligning genomic sequences. Also, Albors et al. (2025) introduces a novel framework for training genomic language models, a type of LLM, that integrates phylogenetic tree data directly into the model’s training process, enhancing its ability to predict functionally disruptive variants.

In summary, despite the ongoing incorporation of new data types and development of methodologies (Mo et al. 2024), ML is still progressing towards replacing traditional methods of phylogeny reconstruction and related phylogenetic tasks. Some of the main challenges described below.

## Challenges in Phylogenetic DL

### Simulating Phylogenetic Data with Realism

Simulated data are pivotal for evaluating inference and training algorithms. We typically use probabilistic models of sequence evolution to simulate MSAs, with many phylogenetic packages (e.g. Fletcher and Yang 2009) capable of performing this task. However, simulated data often fail to accurately represent empirical data. Model misspecification can be particularly problematic for DL, as algorithms trained on nonrepresentative data lead to poor performance in classification or regression tasks when the discrepancies are severe (e.g. Hejase et al. 2021; Mo and Siepel 2023).

Trost et al. (2023) trained CNN-based classifiers that could easily discriminate between simulated and empirical MSAs. The classifiers had prediction accuracy between 93.0–99.9%, even when applied to highly parameterized models of evolution, including rate variation, invariant sites, and amino acid profiles. This study demonstrates that standard simulators remain simplistic. In particular, the authors show that indels need better modeling and that empirical data usually exhibit a more erratic distribution of site patterns, which standard simulators fail to replicate. To our knowledge, no ML-based simulator has yet been developed that successfully closes this gap. However, a study by Booker et al. (2023) demonstrates that GANs can learn the statistical distribution of population genetic alignments, suggesting that GANs may offer a promising strategy for simulating more realistic phylogenetic data.

To address the effects of misspecification, various strategies, such as domain adaptation (DA), can be employed. Unlike conventional DL applications, DA builds models for target datasets with distributions different from the source training data (Wilson and Cook 2020). Domain-adapted NNs aim to learn a domain-invariant representation of the data through a feature extractor NN, which minimizes domain divergence. Mo and Siepel (2023) used this method to infer positive selection from ancestral recombination graph features and estimate recombination rates from genotype matrices. This framework facilitated the detection of sweeps and the estimation of selection coefficients and recombination rates. Although these applications are not phylogenetic, they share the common characteristic of nonindependent sequences with a shared history. Therefore, we foresee the potential to extend the DA framework to phylogenetic applications.

### Considerations for Hyperparameter Optimization in DL for Phylogenetics

DL algorithms are increasingly being applied in phylogenetic inference, however, these applications often lack hyperparameter optimization. Hyperparameters are those that are not estimated during the learning phase and must be set before training (Yang and Shami 2020). Examples include the activation function, number of layers and nodes in a NN. Traditionally, hyperparameters have been tuned manually, but this approach is highly ineffective due to the large and diverse hyperparameter space, which includes categorical, continuous, and discrete variables, as well as their interactions. Without automated hyperparameter tuning, we cannot be confident that our models are performing optimally.

Automatic hyperparameter optimization methods have been developed to address this issue. Bayesian optimization is one such method that determines the next hyperparameter values based on previous ones, thus avoiding many unnecessary evaluations (Eggensperger et al. 2013). However, because Bayesian optimization operates sequentially, it cannot be easily parallelized. Alternative approaches, such as grid search (Bergstra et al. 2011), random search (James 2012), and many others reviewed by Yang and Shami (2020) and Bischl et al. (2023), identify the best-performing parameter combinations by extensively testing within a defined search space. Although these methods may be limited by execution time, novel application programming interfaces (e.g. Optuna by Akiba et al. 2019) combined with parallelized coding enable efficient hyperparameter optimization in reasonably sized parameter spaces.

Recently, methods have been developed specifically to optimize the hyperparameters of NNs (Jin et al. 2019; Zimmer et al. 2021). We advocate for a broader adoption of hyperparameter optimization methods in phylogenetics, when computational resources allow. This is not only because they enhance the performance of DL models but also because they promote the consistency in inferred architectures across studies with similar datasets, based on the idea that similar model architectures are likely to emerge under similar conditions.

### Assessing the Robustness of DL Predictions

Well established methods in phylogenetics, such as Bayesian methods and maximum likelihood, offer strategies for measuring the degree of uncertainty in the inferred parameters. Assessing this uncertainty is fundamental, as it forms the basis for hypothesis testing and model selection. DL methods do not offer a natural way to determine this uncertainty in the data.

Although the measurement of uncertainty is not inherent to machine learning methods, they can be modified to assist us in this process. Voznica et al. (2022) use a form of parametric bootstrap to estimate 95% confidence intervals for their neural network estimators. Given that this process is computationally demanding, attempts have been made to reduce the computational cost, for example, using a dropout method (Gal and Ghahramani 2016). In contrast, Thompson et al. (2024) applied a technique known as conformalized quantile regression (CQR) as part of a CNN training procedure to obtain calibrated probability intervals in a phylodynamic estimation task. CQR is a statistical method that combines quantile regression with conformal prediction to provide prediction intervals that adapt to varying levels of uncertainty in the data (Romano et al. 2019). Notably, the authors demonstrate that their predicted intervals greatly overlap with the Bayesian highest posterior density intervals but have lower precision. Strategies for establishing branch support (e.g. in bootstrapping) have also been proposed. Ecker et al. (2024) developed an approach that combined DL methods with already fast approximate likelihood ratio tests for estimating branch support values. These studies show that DL methods can be modified or combined with traditional methods to access uncertainty and potentially be integrated into statistical decision tasks in the future.

## Conclusions and Future Perspectives

DL methods have demonstrated capability in reconstructing four-taxon and larger trees in notoriously difficult scenarios, such as the presence of indels (Suvorov et al. 2020), complex model spaces (Smith and Hahn 2023; Nesterenko et al. 2025), and likelihoods with multiple optima (Azouri et al. 2024). However, they have not yet surpassed traditional maximum likelihood and Bayesian techniques in terms of accuracy and versatility in all relevant scenarios. Nevertheless, the potential of AI to speed up processes and conserve computational resources, which are commonly consumed by traditional methods like model selection (Voznica et al. 2022) and estimating branch support values (Ecker et al. 2024), is extremely encouraging.

Progress in population genetics has advanced rapidly, allowing some methods to be transferred to phylogeny. In particular, given the vast amounts of data currently being generated, there is a growing need for unified frameworks that can integrate both interspecific and intraspecific variation. There are examples of DL applications for species delimitation (Perez et al. 2022), however, to the best of our knowledge, DL has not been applied to phylogenetic reconstruction at both timescales. While likelihood-based approaches have already been developed (De Maio et al. 2013; Borges et al. 2019, 2022; Braichenko et al. 2024), these methods could greatly benefit from the scalability and speed offered by DL techniques. DL methods typically handle statistical dependencies well, as demonstrated by some population genetics applications that account for linkage or haplotype structure (e.g. Hejase et al. 2021). Similar approaches could therefore be explored to address neighboring dependencies in phylogenetics. Prillo et al. (2023) have shown that phylogenetic substitution models which allow for nearest neighbor effects, can be quickly estimated. LLM architectures can consider dependencies not only to adjacent neighbors but also across entire chromosomes or genomes (see Benegas et al. 2023; Ecker et al. 2024; Nesterenko et al. 2025).

Population genetics is starting to benefit from explainable AI, which allows researchers to interpret complex biological data with more clarity (Riley et al. 2024). However, this aspect is still absent in phylogenetic DL. Furthermore, demography (Wang et al. 2021) and protein structure (Moi et al. 2023; Nagar et al. 2023), could enhance phylogenetic frameworks by providing additional insights to better disentangle evolutionary histories. We expect these to be promising avenues for future development.

## Data Availability

No new data sets were generated or analyzed in support of this perspective.
